# Endobiliary Radiofrequency Ablation in Cholangiocarcinoma: Current Evidence and Future Perspectives

**DOI:** 10.3390/jcm15155781

**Published:** 2026-07-23

**Authors:** Razan Aburumman, Muhammad Saad Faisal, Sumit Singla

**Affiliations:** 1 Department of Internal Medicine, Henry Ford Hospital, Detroit, MI 48202, USA; 2 Division of Gastroenterology and Hepatology, Henry Ford Hospital, Detroit, MI 48202, USA

**Keywords:** cholangiocarcinoma, malignant biliary obstruction, endobiliary radiofrequency ablation, RFA

## Abstract

Cholangiocarcinoma (CCA) is an aggressive malignancy associated with malignant biliary obstruction and it has poor overall survival. Most patients present with unresectable disease which limits curative treatment options and highlights the need for effective palliative therapies. Endobiliary radiofrequency ablation (RFA) has emerged as a minimally invasive technique aimed at reducing intraductal tumor burden, improving biliary drainage, and potentially prolonging survival. Recently, multiple randomized controlled trials, retrospective studies, and meta-analyses have evaluated the role of endobiliary RFA in patients with CCA, with mixed results. Available evidence suggests that RFA may provide a survival benefit in selected patients. However, its effects on stent patency and quality of life remain inconsistent across studies. Current data also demonstrate an overall acceptable safety profile. In this review, we summarize the procedural techniques, currently available evidence, adverse events, patient selection considerations, guideline recommendations, and future directions regarding the use of endobiliary RFA in CCA.

## 1. Introduction

Cholangiocarcinoma (CCA) is a rare tumor that originates from cholangiocytes in the bile ducts and is a leading cause of malignant biliary obstruction [1,2]. CCA is the second-most common primary liver tumor, with an incidence rate of 0.3–6 per 100,000 individuals per year [3]. It has a poor prognosis rate, with a five-year survival of 7–20% [4,5]. Most deaths from CCA are attributable to local disease progression, supporting the use of locoregional therapies, while avoiding some of the side effects of systemic treatments [6]. Traditionally, surgical resection is thought to be the only potentially curative option for patients with CCA [7]. However, most patients present at a late stage for curative surgical resection, or comorbidity precludes the radical surgery necessary [8].

Preoperative or palliative biliary drainage with stent placement, performed through endoscopic retrograde cholangiopancreatography (ERCP) or percutaneous transhepatic cholangiography (PTC), plays an important role in the management of extrahepatic CCA. Stenting is important as it has been shown to relieve the jaundice and pruritus and improve the quality of life [9,10]. However, even though self-expandable metal stents (SEMS) have a lower occlusion rate than plastic stents, SEMS have an occlusion rate of 19–40% due to epithelial hyperplasia, tumor ingrowth and overgrowth, dislocation, and debris and clot accumulation [11]. In the ongoing effort to address SEMS occlusion, several strategies have been explored, including covered stents, modified stent designs, and biliary intraluminal irradiation stents. However, these approaches have shown limited success in meaningfully extending SEMS patency duration [12].

In recent years, endobiliary radiofrequency ablation (RFA) has emerged as a promising local therapeutic modality for the management of malignant biliary obstruction [13]. Endobiliary RFA is used mainly as a palliative technique to reduce intraductal tumor burden, improve biliary drainage, and potentially prolong stent patency [14,15]. It is also hypothesized that injury to the tumor and tumor-associated vasculature, with subsequent dissemination of tumor fragments into the circulation, can stimulate an immune response against the malignancy [16]. This review provides an updated synthesis of the current evidence of the role of endobiliary RFA in the management of CCA.

## 2. Methods

A narrative literature review was performed using PubMed/MEDLINE to identify studies evaluating endobiliary RFA in CCA. The search included publications through April 2026 using combinations of the terms cholangiocarcinoma, malignant biliary obstruction, radiofrequency ablation, endobiliary radiofrequency ablation, ERCP, stent, and photodynamic therapy. Priority was given to randomized controlled trials, systematic reviews, meta-analyses, guideline statements, and large observational studies. Additional relevant references were identified through manual review of the cited literature.

## 3. Procedural Technique

RFA is a minimally invasive technique that works by delivering high-frequency alternating electrical current through an electrode, which generates heat within the targeted tissue, and facilitates coagulative necrosis of affected tumor cells [15]. The thermal injury typically extends 3–8 mm from the probe and depends on the amount of energy applied. RFA can be performed using monopolar or bipolar probes with tissue destruction occurring through frictional heat generated by ionic movement within the tumor. Effective ablation usually requires temperatures of 60–80 °C maintained for 1–2 min at 7–10 W which results in protein denaturation, cell dehydration, and tumor cell death while minimizing damage to surrounding healthy tissue [17]. Local thermal ablation aims to decrease tumor burden and limit intraductal tumor progression with the primary goals of prolonging stent patency by reducing tumor ingrowth and hyperplastic tissue formation, while also potentially improving survival outcomes. Maintaining biliary drainage for longer periods helps prevent recurrent jaundice and cholangitis, supports uninterrupted systemic therapy, and contributes to improved overall clinical status [18].

Currently, two devices are available for intrabiliary RFA: the Habib HPB-RF probe (Boston Scientific Corp., Marlborough, MA, USA) and the ELRA RF catheter (Taewoong Medical, Gyeonggi-Do, South Korea) [19]. They are both bipolar endobiliary RFA catheters delivered over a guidewire under fluoroscopic guidance. The ELRA system incorporates real-time temperature and impedance feedback enabling controlled and consistent ablation and minimizing thermal injury. In contrast, the Habib catheter relies on preset power and time settings without real-time tissue feedback [19]. There are currently no studies comparing the efficacy and safety of these two devices.

RFA can be performed either by an endoscopic or percutaneous approach [18]. Both approaches use the same endobiliary RFA probe to deliver thermal energy, but they differ in access route and the volume of supporting evidence. In the endoscopic approach, the radiofrequency application is performed through endoscopic retrograde cholangiopancreatography (ERCP). In the percutaneous approach, RFA is performed by advancing the RFA catheter through an existing percutaneous transhepatic biliary drainage (PTBD) tract [20]. The endoscopic approach has been more widely studied. However, a recent retrospective analysis of eight patients with unresectable intrahepatic/perihilar CCA who underwent percutaneous endobiliary RFA showed that this approach is safe and technically feasible with a 100% technical success rate and is associated with favorable laboratory and clinical outcomes [20].

There are no head-to-head trials directly comparing endoscopic versus percutaneous RFA that currently exist. However, the advantages of each approach are largely inferred from the broader literature comparing endoscopic versus percutaneous biliary interventions. The percutaneous approach avoids post-ERCP pancreatitis (PEP) which remains one of the main adverse events associated with endoscopic interventions [21]. Also, the endoscopic approach utilizes the body’s natural orifice and avoids the need for an external drain, thereby reducing drain-related discomfort, maintenance issues, and catheter-associated complications. Endoscopic access also avoids hepatic parenchymal puncture, which may decrease the risk of bleeding and hemorrhagic complications seen with percutaneous approaches [22]. In addition, endoscopy offers the advantage of simultaneous tissue acquisition through brushings, biopsies, or cholangioscopy-directed sampling during the same session. However, ERCP can be technically challenging in patients with altered anatomy, like in patients with Roux-en-Y gastric bypass (RYGB) [23]. The choice between endoscopic and percutaneous RFA should therefore be individualized based on biliary anatomy, tumor location, prior interventions, and the feasibility of achieving adequate biliary access.

## 4. Current Evidence

Since the first pilot study completed in 2011 [24], the evidence base for endobiliary RFA has grown substantially over the last few years, but it remains somewhat heterogeneous. The overall data support RFA as a safe adjuvant palliative modality, though its effect on survival remains debated. Although this review focuses on CCA, it is important to recognize that much of the available evidence includes mixed malignant biliary obstruction populations, including pancreatic, ampullary, and other biliary malignancies. These studies are discussed when they provide clinically relevant information but should be interpreted with this limitation in mind.

### 4.1. Randomized Controlled Trial Evidence

A multicenter randomized controlled trial (RCT) in China was completed by Gao et al. [25], including patients with locally advanced or metastatic CCA or ampullary cancer who were unsuitable for surgery (*n* = 174), comparing RFA plus plastic stenting with plastic stenting alone. This study reported a significantly improved median overall survival (OS) in the RFA group (14.3 vs. 9.2 months, HR 0.49, *p* < 0.001) with no difference in stent patency [25].

However, an European RCT (*n* = 161) comparing endoluminal RFA plus stenting with stenting alone in patients with malignant biliary obstruction was stopped early for futility, showing no difference in survival for CCA (10.5 vs. 10.6 months, *p* = 0.58), stent patency, or quality of life [26]. Similarly, a German multicenter RCT (*n* = 86) comparing SEMS only or RFA followed by SEMS found no benefit on patency rate or survival [27]. Another single-center RCT from Thailand that included 39 patients with unresectable perihilar CCA who were randomized to receive RFA + SEMS or SEMS alone found no significant difference in stent patency (71 vs. 78 days, *p* = 0.809) or OS (94 vs. 79 days, HR 1.31, 95% CI 0.66–2.58, *p* = 0.735) [28].

The conflicting data might be related to variability in stent type, baseline disease burden and prognosis, RFA technique, and patient selection. In the study by Gao et al. [25], plastic stents were used with two scheduled interventions around 3 months apart. Plastic stents allow re-access to the biliary tree, which makes repeated ablation sessions technically feasible, helping to maintain ductal patency [18]. However, the huge variability in the mentioned trial designs and follow-up limits direct comparability across trials.

### 4.2. Meta-Analytic Data

Many meta-analyses have been published evaluating RFA plus stenting versus stenting alone for malignant biliary obstruction, with varying inclusion criteria.

Some meta-analyses included only RCTs. Balducci et al. (2024) [14] included only RCTs in their meta-analysis that studied patients with inoperable extrahepatic CCA, treated with RFA plus stenting versus stenting alone. Five randomized trials and 370 patients were included. They found a non-significant trend toward improved OS (HR 0.62, *p* = 0.09) with high heterogeneity (I^2^ = 80%), but a significant benefit in the plastic stent subgroup (HR 0.42, *p* = 0.009) and improved stent patency overall (HR 0.64, *p* = 0.01) [14]. However, another newer 2025 meta-analysis of nine RCTs by Ramai et al. that included 750 patients with any malignant biliary obstruction found that patients with CCA experienced an OS benefit with RFA plus stenting compared with stenting alone (*p* < 0.001); however, stent patency was unaffected (*p* = 0.08) [29].

Other meta-analyses included RCTs and observational studies. A meta-analysis by Zhou et al. [30], published in 2025, that focused on hilar CCA (11 studies, 874 patients) showed a pooled OS HR of 0.74 (95% CI 0.61–0.89, *p* = 0.002) and improved stent patency (HR 0.77, *p* = 0.03) with RFA, with comparable adverse events. Also, the survival benefit was more pronounced when RFA was combined with chemotherapy (HR 0.57) [30]. Hayat et al. [31], in 2024, published a meta-analysis that included studies comparing RFA with stenting versus stenting alone for treating any malignant biliary strictures. A total of 13 studies with 1339 patients were identified. They reported a mean OS difference of 90.5 days (95% CI 49.0–132.1) favoring RFA and a mean stent patency difference of 43.5 days (95% CI 25.6–61.4) also favoring RFA [31]. Tarar et al. [32] in 2023, published another meta-analysis that included studies measuring the effect of RFA plus stents compared to stent placement only in malignant biliary obstruction. Their study included a total of 17 studies (14 observational and 3 RCTs) containing 1766 patients. The weighted pooled mean survival difference was 58.5 days in favor of the RFA treatment group, and the mean difference in stent patency was better in the RFA plus stent group by 45.3 days [32].

Taken together, despite some variability in statistical significance, the overall direction of evidence across most RCTs and meta-analyses suggests that RFA combined with stenting may improve survival, with a more modest and less consistent effect on stent patency. One major reason is the difference in included study designs. The mentioned meta-analyses also differed in their inclusion criteria for malignant biliary obstruction etiologies of the studies they included, which introduces variability in tumor biology, expected survival, and response to biliary interventions. It is important to note that studies also differed in how they measured stent patency and survival outcomes. Definitions of stent dysfunction, timing of follow-up, repeat interventions, and stent types were not uniform across studies.

### 4.3. Combination with Chemotherapy

A retrospective cohort study of 66 patients with advanced extrahepatic CCA found that RFA combined with chemotherapy significantly prolonged median OS (17.3 vs. 8.6 months, *p* = 0.004) and progression-free survival (12.9 vs. 5.7 months, *p* = 0.045) compared with chemotherapy alone, and had comparable hematologic toxicity but it was associated with increased risk for cholangitis [33]. This suggests a potential synergistic effect between RFA and systemic therapy.

Overall, these findings suggest that while RFA may have a survival advantage in selected patients, its impact on stent patency and clinical outcomes remains inconsistent across studies. Further large-scale, well-designed trials are needed to better define the patient populations most likely to benefit and to standardize treatment protocols.

### 4.4. Comparison with Photodynamic Therapy

Photodynamic therapy (PDT) is a non-thermal, localized ablative technique that works by combining a light-sensitive drug with targeted light exposure to destroy malignant cells. It is typically a well-tolerated therapy with a reported favorable side effect profile and is considered a strong option for palliative therapy in CCA [15].

A retrospective analysis of forty-eight patients with unresectable CCA who underwent ERCP-directed ablative therapy for palliation of unresectable CCA using ERCP-directed RFA or PDT showed that there was no significant difference in median OS between RFA and PDT (9.6 months versus 7.5 months, *p* = 0.799). However, RFA-treated patients experienced more stent occlusion (*p* = 0.008) and risk of cholangitis (*p* = 0.008) [34]. Another retrospective study of 63 with extrahepatic CCA found similar survival for RFA and chemotherapy (13.8 months) and PDT and chemotherapy (12.7 months) with a non-significant trend toward the best outcomes in patients receiving both modalities (20.2 months, *p* = 0.112) [35].

A large meta-analysis published in 2022 that included a total of 55 studies (2146 patients) reported a pooled survival of 11.9 months with PDT, 8.1 months with RFA, and 6.7 months with stent-only palliation. PDT also demonstrated superior stent patency (6.1 months) and lower 30-day mortality (3.3%) [36]. Of note, it has been reported that in patients with CCA the combination of PDT and chemotherapy was well-tolerated and resulted in significantly longer survival than chemotherapy alone (*p* = 0.022) [37].

Despite the mentioned benefits, PDT requires a specialized treatment workflow and is associated with modality-specific limitations, like requiring post-procedural photosensitivity precautions. Endobiliary RFA, on the other hand, offers practical advantages like the ease of integration into standard endoscopic or percutaneous workflows and the potential for reopening of occluded stents. In addition, RFA is typically less resource-intensive and does not cause post-procedural photosensitivity [20].

### 4.5. Guideline Recommendations

The 2023 American College of Gastroenterology (ACG) Clinical Guideline on Biliary Strictures recognizes a potential survival benefit of RFA in patients with malignant perihilar strictures due to CCA. For patients who are not candidates for surgical resection or liver transplantation, the guideline recommends adjuvant endobiliary ablation (PDT or RFA) in combination with plastic stent placement rather than stenting alone (conditional recommendation, low-quality evidence). However, it emphasizes that the overall quality and strength of evidence are stronger for PDT than for RFA in this setting [38].

## 5. Adverse Events

Endobiliary RFA in CCA has a generally favorable safety profile, with overall adverse event rates comparable to those with stenting alone [30,39]. In a large retrospective series of 120 patients with malignant biliary obstruction (MBO) treated with endoscopic RFA, the overall early adverse event rate was 16.6%, with biliary infection being the most common adverse [40]. Table 1 summarizes the most commonly reported adverse events.

Khizar et al. reported that type 2 diabetes mellitus, bile duct stricture length longer than 2.5 cm, segmental RFA, and single-stent drainage (as opposed to multiple-stent drainage) were associated with increased risk of adverse events in patients with MBO treated with endoscopic RFA [40].

The evidence on quality of life after RFA is limited. In the previously mentioned multicenter RCT by Gao et al., RFA plus plastic stenting resulted in significantly higher Karnofsky performance scores compared to stenting alone, with the benefit persisting up to 9 months post-procedure (all *p* < 0.001) [25]. Another retrospective study by Wu et al. using the FACT-Hep questionnaire (Functional Assessment of Cancer Therapy–Hepatobiliary) found that patients who underwent percutaneous intraductal RFA with stenting had higher functional well-being scores and total FACT-Hep scores during post-procedure follow-up compared to stenting alone [43].

## 6. Patient Selection

The ACG recommends the use of adjuvant endobiliary ablation, including RFA plus plastic stent placement over plastic stent placement alone, in patients with a malignant perihilar stricture due to CCA who are not candidates for resection or transplantation. ACG also discussed that RFA should only be initiated after multidisciplinary evaluation to confirm inoperability as it causes a full-thickness injury, which may lead to tissue edema and scarring. This, in turn, will confound subsequent preoperative imaging and complicate decision-making around surgical candidacy [38].

Regarding disease characteristics, Bismuth type III and IV hilar CCA involve more complex biliary strictures and pose greater technical challenges for endoscopic intervention; consequently, these patients have often been excluded from prior studies evaluating RFA [25,44]. Regarding disease stage, RFA is reported to improve survival in cases without distant metastasis. A large-scale retrospective study including 883 patients reported that in patients with CCA, RFA treatment could only improve the prognosis of the CCA without distant metastasis (11.5 months vs. 7.4 months, *p*  <  0.001), and that once distant metastasis occurred, no significant survival benefit was observed in these patients (7.3 months vs. 4.3 months, *p* = 0.182) [45]. In addition, patients planned for systemic treatment may have benefit from the addition of RFA. The integration of RFA with chemotherapy has been thought to have a synergistic advantage as RFA facilitates local tumor management while chemotherapy targets systemic disease dissemination. An RCT comparing patients treated with RFA plus chemotherapy to those receiving RFA alone showed that the combination therapy significantly enhances survival [46]. More studies are needed to clearly define the criteria of patients who will benefit the most from RFA.

## 7. Future Directions

Although the evidence supporting endobiliary RFA continues to grow, several important knowledge gaps remain. Head-to-head randomized controlled trials comparing RFA with PDT and other locoregional therapies are still lacking. Additional studies are also needed to define better the long-term safety and cost-effectiveness of RFA. Future research should focus on identifying the patients most likely to benefit from RFA, including the influence of tumor location and disease stage. Studies are also needed to establish standardized procedural protocols, including optimal energy settings, stent selection, and the role of repeat RFA sessions. In addition, further investigation is warranted to determine the optimal integration of RFA with systemic therapy, including the timing of treatment relative to chemotherapy and immunotherapy and whether combination strategies improve clinical outcomes.

Some of the ongoing clinical trials include the ACTICCA-2 trial (NCT06174845), a multicenter, randomized, controlled trial to assess the efficacy and safety of adding biliary RFA to bile duct stenting in patients with CCA receiving palliative systemic treatment [47]. In addition, there is the Ablatio-Bilica trial (NCT06274879), a phase II RCT specifically assessing the safety of combining RFA with immune checkpoint inhibitor-based regimens, a combination hypothesized to be synergistic given RFA-induced immunogenic response against tumor cells [48].

## 8. Conclusions

Endobiliary RFA represents a promising adjunctive palliative therapy in the management of CCA, particularly for patients with unresectable disease and malignant biliary obstruction. Available data suggest a survival benefit in selected patients, but data remain heterogeneous. Also, stent patency and quality of life after have not been uniformly demonstrated. Although multiple studies have proved the safety of RFA, it has been associated with multiple complications which require careful patient selection and procedural planning. Current guidelines support its use in combination with stenting in non-surgical candidates. Large-scale randomized trials are needed to define the optimal role of RFA better. Future studies should focus on studying ideal patient selection, timing relative to systemic therapy, and combination strategies with emerging treatments such as immunotherapy.

## Figures and Tables

**Table 1:** Adverse events related to the use of RFA in the management of malignant biliary strictures.

**Cholangitis/biliary infection**	Reported to be the most common complication occurring in approximately 10.8% of patients in one large series [40]. Cholangitis was significantly more frequent when RFA was combined with systemic chemotherapy (*p* = 0.031) [33].
**Post-procedural abdominal pain**	One meta-analysis that included 9 studies and 505 patients found a significantly higher rate of abdominal pain with RFA compared to stenting alone (31% vs. 20%, *p* = 0.003) [41].
**Hemobilia**	Reported in approximately 4% of cases per the ACG guideline. Thought to be due to hepatic artery pseudoaneurysm formation given the proximity of the biliary confluence to the hepatic vasculature [38].
**Acute pancreatitis**	A meta-analysis reported no statistically significant difference risk of pancreatitis compared to stenting alone (OR 1.03, 95% CI 0.19–5.50) [30].
**Cholecystitis**	A meta-analysis of nine RCTs involving 750 subjects with MBO found a notably increased incidence of cholecystitis with RFA plus stenting vs. stenting alone (5.1% [95% CI 3.1–7.8%] vs. 0.3% [95% CI 0.01–1.5%], respectively) [29].
**Bile duct perforation**	A rarely reported event in practice. An RCT using a temperature-controlled catheter and a large retrospective series reported no cases of perforations [40,42].
**Liver abscess**	Infrequently reported, with no significant difference between RFA and stent-only groups in pooled analysis of 874 patients (OR 0.62, 95% CI 0.07–5.24) [30].
**Vascular complications**	A theoretical but rarely reported adverse event that depends on proximity of tumor to vascular structures.

## Data Availability

No new data were created or analyzed in this study. Data sharing is not applicable to this article.
